# Anesthesia and surgery induce cognitive dysfunction in elderly male mice: the role of gut microbiota

**DOI:** 10.18632/aging.101871

**Published:** 2019-03-23

**Authors:** Gaofeng Zhan, Dongyu Hua, Niannian Huang, Yue Wang, Shan Li, Zhiqiang Zhou, Ning Yang, Riyue Jiang, Bin Zhu, Ling Yang, Fan Yu, Hui Xu, Chun Yang, Ailin Luo

**Affiliations:** 1 Department of Anesthesiology, Tongji Hospital, Tongji Medical College, Huazhong University of Science and Technology, Wuhan, China; 2 Department of Internal Medicine, The Third Affiliated Hospital of Soochow University, Jiangsu, China; * Equal contribution

**Keywords:** cognitive dysfunction, gut microbiota, anesthesia, surgery, aging

## Abstract

It is well known that the incidence of postoperative cognitive dysfunction (POCD) is high in elderly patients. The pathogenesis and therapeutic mechanisms of POCD, however, have not yet been completely elucidated. The effects of gut microbiota, particularly in terms of regulating brain function, have gradually attracted increasing attention. In this study, we investigated the potential role of gut microbiota in POCD in aged male mice and attempted to determine whether alterations in gut microbiota would be helpful in the diagnosis of POCD. POCD and non-POCD mice were classified by hierarchical cluster analysis of behavioral results. Additionally, α- and β-diversity of gut microbiota showed a differential profile between the groups. In total, 24 gut bacteria were significantly altered in POCD mice compared with those in non-POCD mice, in which 13 gut bacteria were significantly correlated with escape latency in the Morris water maze test (MWMT). Remarkably, receiver operating characteristic curves revealed that the *Dehalobacteriaceae* family and *Dehalobacterium* genus are potentially important bacteria for the diagnosis of POCD. These findings indicate that alterations in the composition of gut microbiota are probably involved in the pathogenesis of POCD in aged mice. Novel therapeutic strategies regulating specific gut bacteria may be helpful for the prevention and treatment of POCD.

## Introduction

Cognitive dysfunction after anesthesia and surgery has gradually attracted increasing attention. With an incidence of 23% in elderly patients postoperatively, it places a heavy burden on the patients’ families and society [1–4]. Several lines of evidence show that the high incidence of postoperative cognitive dysfunction (POCD) in elderly patients may be associated with an imbalanced inflammatory response, microcirculation disorder, microembolism formation, and abnormally activated microglia [5–8]. However, therapeutic strategies targeting anti-inflammation, improvements in intracerebral vascular circulation, inhibition of microembolism formation, and regulation of activated microglia have not led to satisfactory clinical results [9,10]. Thus, exploring the exact mechanisms of POCD and developing effective treatments are of great importance.

The gut microbiota refers to the large number of microorganisms in the digestive tract [11–14]; approximately 100 trillion bacteria live in the gut of the human body [15]. Various bacteria are capable of synthesizing vitamins necessary for human growth and development, and these bacteria also have the physiological potential to synthesize amino acids, participate in sugar and protein metabolism, and promote the absorption of mineral elements [16–18]. An increasing number of studies are concerned with the gut microbiota remotely regulating the functions of the central nervous system (CNS) through the vagus nerve or glucagon-like peptide-1 signaling pathway [19–21]. An abnormal composition of the gut microbiota has been observed to be greatly associated with the onset of autism, depression, schizophrenia, and Alzheimer's disease [11,22–25]. Consequently, these results suggest that the gut microbiota, at least partially, is associated with the pathogenesis of CNS diseases; thus, regulating its composition and improving its physiological functions would be beneficial for the prevention and treatment of brain diseases.

To date, the role of gut microbiota in POCD has not yet been clearly determined. Here, we employed 16S rRNA sequencing to observe and compare gut microbiota composition in mice with POCD and non-POCD phenotypes, and we attempted to elucidate whether the gut microbiota plays a critical role in the pathogenesis of POCD.

## RESULTS

### Open field test and Morris water maze test results between the non-POCD and POCD groups

**Figure 1:**
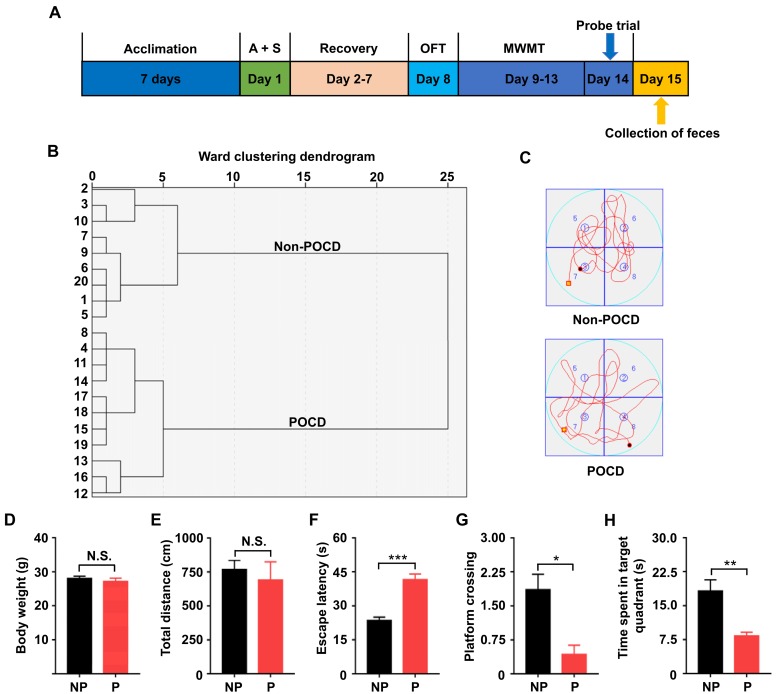
**Comparisons of OFT and MWMT between the non-POCD and POCD groups.** (**A**) The schedule of the present study. Seven days after acclimation, A + S was performed. OFT was performed on day 8 after 6 days of recovery. On days 9–14, mice were scheduled for the MWMT, and the probe trial was performed on day 14. On day 15, fecal samples were collected for 16S rRNA gene sequencing. (**B**) Dendrogram of hierarchical clustering analysis. A total of 20 mice were categorized into non-POCD and POCD groups based on MWMT results of the hierarchical clustering analysis. (**C**) Representative trace graphs of non-POCD and POCD mice in the MWMT. (**D**) Body weight (t = 0.7618, *P* >.05). (**E**) Total distance in OFT (t = 0.5285, *P* >.05). (**F**) Escape latency (t = 6.227, *P* <.001). (**G**) Platform crossing (t = 3.612, *P* <.05). (**H**) Time spent in target quadrant (t = 3.897, *P* <.01). A + S: anesthesia and surgery; MWMT: Morris water maze test; NP: non-POCD; N.S.: not significant; OFT: open field test; P: POCD. Data are shown as mean ± S.E.M. (n = 7). *P <.05, **P <.01 or ***P <.001.

Non-POCD and POCD mice were categorized by hierarchical cluster analysis of escape latency, platform crossing, and time spent in a target quadrant (Figure 1B). Locomotor activity was assessed by the total distance traveled in the open field chamber for 5 min of exploration. The results showed no changes in body weight between the groups (Figure 1D). Additionally, no significant difference was found in total distance traveled between the groups (Figure 1E), indicating that locomotor activity was similar between both groups. The Morris water maze test (MWMT) was used to evaluate cognitive behavior in the two groups. POCD mice exhibited significantly increased escape latency (Figure 1F). In the probe trial, platform crossing times and time spent in a target quadrant were both significantly decreased in POCD mice compared with those in non-POCD mice (Figure 1G and 1H).

### α-diversity and β-diversity of the gut microbiota in non-POCD and POCD mice

**Figure 2:**
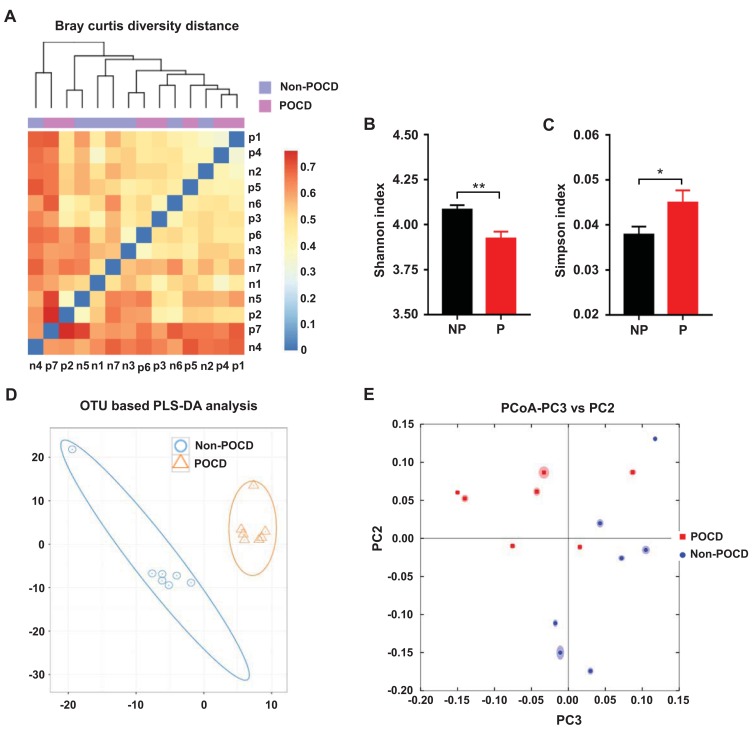
**Differential profiles of the gut microbiota between the non-POCD and POCD groups.** (**A**) Bray–Curtis diversity distance. (**B**) Shannon index (t = 3.454, *P* <.01). (**C**) Simpson index (t = 2.195, *P* <.05). (**D**) PLS-DA analysis of gut bacteria data. (**E**) PCoA analysis of 7 gut bacteria data (PC3 vs. PC2). α-diversity data are shown as mean ± SEM (n = 7). PCoA: principal coordinate analysis; PLS-DA: partial least squares discrimination analysis. *P <.05, **P <.01.

α-diversity refers to the diversity of species and bacteria within a community or habitat, whereas β-diversity represents the differentiation among habitats [11,26]. Shannon and Simpson indices are commonly used to evaluate the α-diversity of gut microbiota. There was a significant decrease in the Shannon index but a significant increase in the Simpson index in POCD mice compared with non-POCD mice (Figure 2B and 2C). Furthermore, a partial least squares discrimination analysis (PLS-DA) and principal coordinates analysis (PCoA) revealed that the dots of the non-POCD group were clearly separated from that of the POCD group (Figure 2D and 2E). Therefore, it is likely that the composition of the gut microbiota is significantly different between the two groups.

### Abundance of the composition of gut microbiota at phylum, class, order, family, genus, and species levels in the non-POCD and POCD mice

**Figure 3:**
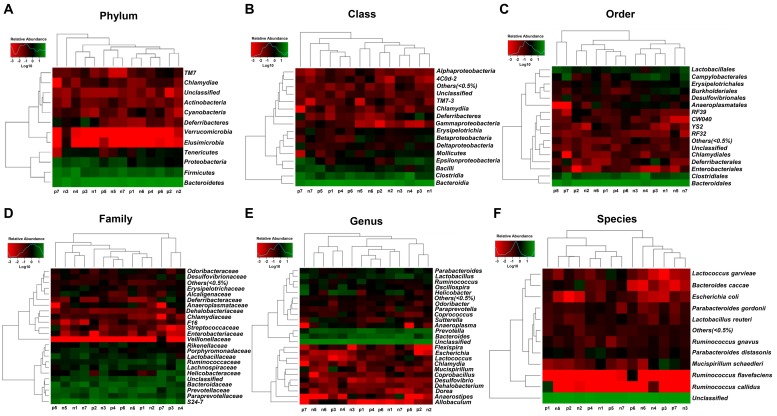
**Heatmaps of the composition of gut bacterium at phylum, class, order, family, genus, and species levels between the non-POCD and POCD groups.** (**A**) Heatmap (phylum level). (**B**) Heatmap (class level). (**C**) Heatmap (order level). (**D**) Heatmap (family level). (**E**) Heatmap (genus level). (**F**) Heatmap (species level).

Heat maps of the gut microbiota composition at the phylum, class, order, family, genus, and species levels in the non-POCD and POCD groups are shown (Figure 3A–3F).

### Alterations in the gut microbiota composition between the POCD and non-POCD mice

**Figure 4:**
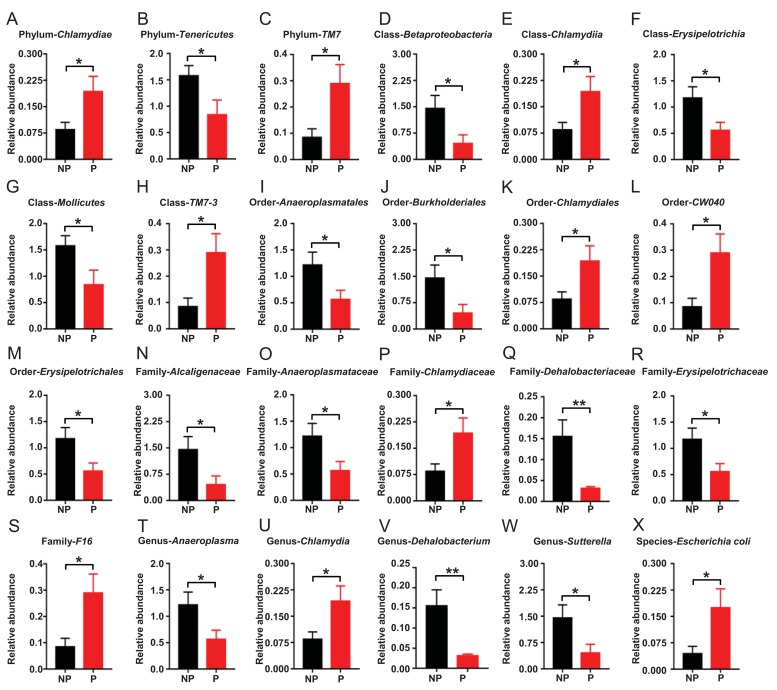
**Differential levels of the gut bacterium between the non-POCD and POCD groups.** (**A**) Relative abundance of Phylum *Chlamydiae* (t = 2.258, *P* <.05). (**B**) Relative abundance of Phylum *Tenericutes* (t = 2.186, *P* <.05). (**C**) Relative abundance of Phylum *TM7* (t = 2.591, *P* <.05). (**D**) Relative abundance of Class *Betaproteobacteria* (t = 2.255, *P* < 0.05). (**E**) Relative abundance of Class *Chlamydiae* (t = 2.258, *P* <.05). (**F**) Relative abundance of Class *Erysipelotrichia* (t = 2.359, *P* <.05). (**G**) Relative abundance of Class *Mollicutes* (t = 2.186, *P* <.05). (**H**) Relative abundance of Class *TM7-3* (t = 2.591, *P* <.05). (**I**) Relative abundance of Order *Anaeroplasmatales* (t = 2.208, *P* <.05). (**J**) Relative abundance of Order *Burkholderiales* (t = 2.255, *P* <.05). (**K**) Relative abundance of Order *Chlamydiales* (t = 2.258, *P* <.05). (**L**) Relative abundance of Order *CW040* (t = 2.591, *P* <.05). (**M**) Relative abundance of Order *Erysipelotrichales* (t = 2.359, *P* <.05). (**N**) Family *Alcaligenaceae* (t = 2.255, *P* <.05). (**O**) Relative abundance of Family *Anaeroplasmataceae* (t = 2.208, *P* <.05). (**P**) Relative abundance of Family *Chlamydiaceae* (t = 2.258, *P* <.05). (**Q**) Relative abundance of Family *Dehalobacteriaceae* (t = 3.118, *P* <.01). (**R**) Relative abundance of Family *Erysipelotrichaceae* (t = 2.359, *P* <.05). (**S**) Relative abundance of Family *F16* (t = 2.591, *P* <.05). (**T**) Relative abundance of Genus *Anaeroplasma* (t = 2.208, *P* <.05). (**U**) Relative abundance of Genus *Chlamydia* (t = 2.258, *P* <.05). (**V**) Relative abundance of Genus *Dehalobacterium* (t = 3.118, *P* <.01). (**W**) Relative abundance of Genus *Sutterella* (t = 2.255, *P* <.05). (**X**) Relative abundance of Species *Escherichia coli* (t = 2.263, *P* <.05).

16S rRNA gene sequencing was used to determine the differences in the composition of gut microbiota between the POCD and non-POCD mice. The results revealed that a total of 24 gut bacteria at six phylogenetic levels (phylum, class, order, family, genus, and species) were significantly altered in fecal samples of mice between the groups (Figure 4A–4X). The relative abundance of 10 bacteria was significantly increased in the POCD group compared with the non-POCD group (Figure 4A, 4C, 4E, 4H, 4K, 4L, 4P, 4S, 4U, and 4X). In contrast, the relative abundance of 14 bacteria was significantly decreased in the POCD group compared with the non-POCD group (Figure 4B, 4D, 4F, 4G, 4I, 4J, 4M, 4O, 4Q, 4R, 4T, 4V and 4W).

### Correlation analysis between escape latency and gut bacteria levels

**Figure 5:**
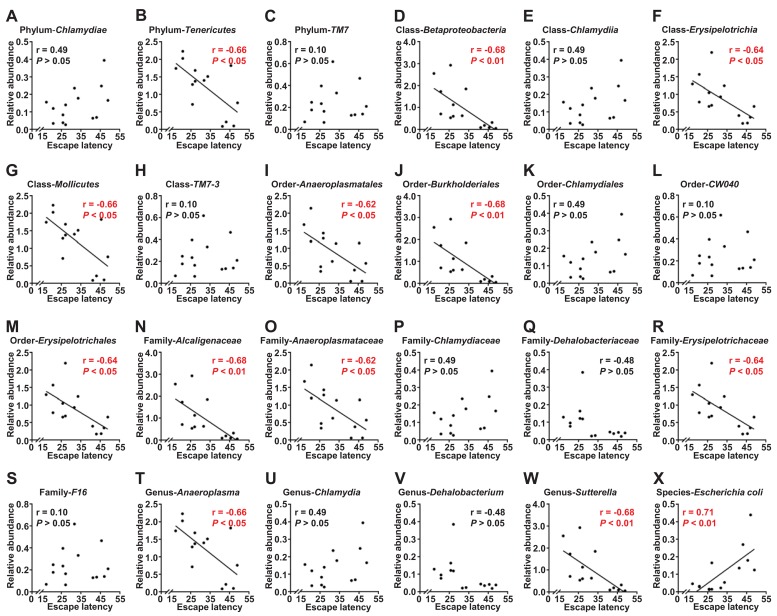
**Correlations between escape latency and the composition of gut bacterium (N=14).** (**A**) Phylum Chlamydiae (r = 0.49, *P*>.05). (**B**) Phylum *Tenericutes* (r = −0.66, *P*<.05). (**C**) Phylum *TM7* (r = 0.10, *P*>.05). (**D**) Class *Betaproteobacteria* (r = −0.68, *P*<.01). (**E**) Class *Chlamydiae* (r = 0.49, *P*>.05). (**F**) Class *Erysipelotrichia* (r = −0.64, *P*<.05). (**G**) Class *Mollicutes* (r = −0.66, *P*<.05). (**H**) Class *TM7* 3 (r = 0.10, *P*>.05). (**I**) Order *Anaeroplasmatales* (r = −0.62, *P*<.05). (**J**) Order *Burkholderiales* (r = −0.68, *P*<.01). (**K**) Order *Chlamydiales* (r = 0.49, *P*>.05). (**L**) Order *CW040* (r = 0.10, *P*>.05). (**M**) Order *Erysipelotrichales* (r = −0.64, *P*<.05). (**N**) Family *Alcaligenaceae* (r = −0.68, *P*<.01). (**O**) Family *Anaeroplasmataceae* (r = −0.62, *P*<.05). (**P**) Family *Chlamydiaceae* (r = 0.49, *P*>.05). (**Q**) Family *Dehalobacteriaceae* (r = −0.48, *P*>.05). (**R**) Family *Erysipelotrichaceae* (r = −0.64, *P*<.05). (**S**) Family *F16* (r = 0.10, *P*>.05). (**T**) Genus *Anaeroplasma* (r = −0.66, *P*<.05). (**U**) Genus *Chlamydia* (r = 0.49, *P*>.05). (**V**) Genus *Dehalobacterium* (r = −0.48, *P*>.05). (**W**) Genus *Sutterella* (r = −0.68, *P*<.01). (**X**) Species *Escherichia coli* (r = 0.71, *P*<.01).

Escape latency in the probe trial was selected to represent MWMT behavior, a reflection of spatial memory [11]. Correlations between the escape latency of a total of 14 mice and the relative abundance of 24 bacteria were analyzed (Figure 5A–5X). The results revealed that 12 gut bacteria were negatively correlated with escape latency (Figure 5B, 5D, 5F, 5G, 5I, 5J, 5M–5O, 5R, 5T, and 5W). In contrast, the relative abundance of *Escherichia coli* (*E. coli*) was positively correlated with escape latency (Figure 5X).

### Evaluation of gut bacteria for the diagnosis of POCD using receiver operating characteristic curve analysis

**Figure 6:**
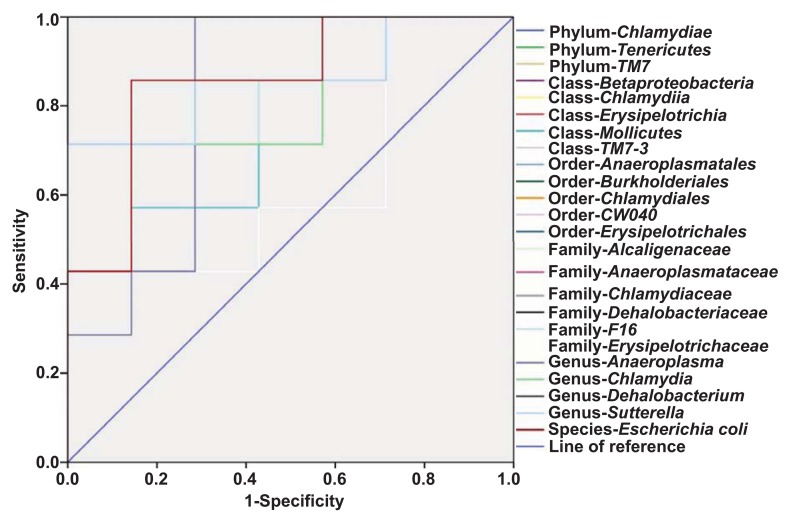
**ROC curves of the gut bacterium count for the diagnosis of anesthesia- and surgery-induced POCD.** (**A**) Phylum *Chlamydiae* (AUC, 0.837). (**B**) Phylum *Tenericutes* (AUC, 0.755). (**C**) Phylum *TM7* (AUC, 0.612). (**D**) Class *Betaproteobacteria* (AUC, 0.857). (**E**) Class *Chlamydiae* (AUC, 0.837). (**F**) Class *Erysipelotrichia* (AUC, 0.857). (**G**) Class *Mollicutes* (AUC, 0.755). (**H**) Class *TM7-3* (AUC, 0.612). (**I**) Order *Anaeroplasmatales* (AUC, 0.816). (**J**) Order *Burkholderiales* (AUC, 0.857). (**K**) Order *Chlamydiales* (AUC, 0.837). (**L**) Order *CW040* (AUC, 0.612). (**M**) Order Erysipelotrichales (AUC, 0.857). (**N**) Family Alcaligenaceae (AUC, 0.857). (**O**) Family *Anaeroplasmataceae* (AUC, 0.816). (**P**) Family *Chlamydiaceae* (AUC, 0.837). (**Q**) Family *Dehalobacteriaceae* (AUC, 1.0). (**R**) Family *Erysipelotrichaceae* (AUC, 0.857). (**S**) Family-*F16* (AUC, 0.612). (**T**) Genus *Anaeroplasma* (AUC, 0.816). (**U**) Genus *Chlamydia* (AUC, 0.837). (**V**) Genus *Dehalobacterium* (AUC, 1.0). (**W**) Genus *Sutterella* (AUC, 0.857). (**X**) Species *Escherichia coli* (AUC, 0.857). A + S: anesthesia and surgery; AUC: area under curve; ROC: receiver operating characteristic.

Receiver operating characteristic (ROC) curves were constructed to indicate the diagnostic ability of the gut bacteria in POCD (Figure 6). The best cutoff values, sensitivity, specificity, positive and negative predictive values, and accuracy of gut bacteria for the diagnosis of anesthesia- and surgery-induced POCD are summarized in Table 1.

**Table 1:** Evaluation of the gut bacterium for the diagnosis of anesthesia- and surgery-induced POCD.

**Evaluation index**	**Cut-off value**	**Sensitivity**	**Specificity**	**Positive predictive value**	**Negative predictive value**	**Accuracy**
**Phylum-*Chlamydiae,* (n)**	**0.1554**	**71.4% (5/7)**	**100% (7/7)**	**100% (5/5)**	**77.8% (7/9)**	**85.7% (12/14)**
**Phylum-*Tenericutes,* (n)**	**0.2099**	**42.9% (3/7)**	**100% (7/7)**	**100% (3/3)**	**63.6% (7/11)**	**71.4% (10/14)**
**Phylum-*TM7,* (n)**	**0.0678**	**100% (7/7)**	**28.6% (2/7)**	**58.3% (7/12)**	**100% (2/2)**	**64.3% (9/14)**
**Class-*Betaproteobacteria,* (n)**	**0.3174**	**71.4% (5/7)**	**100% (7/7)**	**100% (5/5)**	**77.8% (7/9)**	**85.7% (12/14)**
**Class-*Chlamydiia,* (n)**	**0.1554**	**71.4% (5/7)**	**100% (7/7)**	**100% (5/5)**	**77.8% (7/9)**	**85.7% (12/14)**
**Class-*Erysipelotrichia,* (n)**	**0.6544**	**71.4% (5/7)**	**100% (7/7)**	**100% (5/5)**	**77.8% (7/9)**	**85.7% (12/14)**
**Class-*Mollicutes,* (n)**	**0.2099**	**42.9% (3/7)**	**100% (7/7)**	**100% (3/3)**	**63.6% (7/11)**	**71.4% (10/14)**
**Class-*TM7-3,* (n)**	**0.0678**	**100% (7/7)**	**28.6% (2/7)**	**58.3% (7/12)**	**100% (2/2)**	**64.3% (9/14)**
**Order-*Anaeroplasmatales,* (n)**	**1.1459**	**100% (7/7)**	**71.4% (5/7)**	**77.8% (7/9)**	**100% (5/5)**	**85.7% (12/14)**
**Order-*Burkholderiales,* (n)**	**0.3174**	**71.4% (5/7)**	**100% (7/7)**	**100% (5/5)**	**77.8% (7/9)**	**85.7% (12/14)**
**Order-*Chlamydiales,* (n)**	**0.1554**	**71.4% (5/7)**	**100% (7/7)**	**100% (5/5)**	**77.8% (7/9)**	**85.7% (12/14)**
**Order-*CW040,* (n)**	**0.0678**	**100% (7/7)**	**28.6% (2/7)**	**58.3% (7/12)**	**100% (2/2)**	**64.3% (9/14)**
**Order-*Erysipelotrichales,* (n)**	**0.6544**	**71.4% (5/7)**	**100% (7/7)**	**100% (5/5)**	**77.8% (7/9)**	**85.7% (12/14)**
**Family-*Alcaligenaceae,* (n)**	**0.3174**	**71.4% (5/7)**	**100% (7/7)**	**100% (5/5)**	**77.8% (7/9)**	**85.7% (12/14)**
**Family-*Anaeroplasmataceae,* (n)**	**1.1459**	**100% (7/7)**	**71.4% (5/7)**	**77.8% (7/9)**	**100% (5/5)**	**85.7% (12/14)**
**Family-*Chlamydiaceae,* (n)**	**0.1554**	**71.4% (5/7)**	**100% (7/7)**	**100% (5/5)**	**77.8% (7/9)**	**85.7% (12/14)**
**Family-*Dehalobacteriaceae,* (n)**	**0.0436**	**100% (7/7)**	**100% (7/7)**	**100% (7/7)**	**100% (7/7)**	**100% (14/14)**
**Family-*Erysipelotrichaceae,* (n)**	**0.6544**	**71.4% (5/7)**	**100% (7/7)**	**100% (5/5)**	**77.8% (7/9)**	**85.7% (12/14)**
**Family-*F16,* (n)**	**0.0678**	**100% (7/7)**	**28.6% (2/7)**	**58.3% (7/12)**	**100% (2/2)**	**64.3% (9/14)**
**Genus-*Anaeroplasma,* (n)**	**1.1459**	**100% (7/7)**	**71.4% (5/7)**	**77.8% (7/9)**	**100% (5/5)**	**85.7% (12/14)**
**Genus-*Chlamydia,* (n)**	**0.1554**	**71.4% (5/7)**	**100% (7/7)**	**100% (5/5)**	**77.8% (7/9)**	**85.7% (12/14)**
**Genus-*Dehalobacterium,* (n)**	**0.0436**	**100% (7/7)**	**100% (7/7)**	**100% (7/7)**	**100% (7/7)**	**100% (14/14)**
**Genus-*Sutterella,* (n)**	**0.3174**	**71.4% (5/7)**	**100% (7/7)**	**100% (5/5)**	**77.8% (7/9)**	**85.7% (12/14)**
**Species-*Escherichia coli,* (n)**	**0.0456**	**85.7% (6/7)**	**85.7% (6/7)**	**85.7% (6/7)**	**85.7% (6/7)**	**85.7% (12/14)**

## DISCUSSION

The MWMT has been widely employed to assess the symptoms of POCD in rodents [1,11,27]. In the present study, we categorized the mice into POCD and non-POCD groups by hierarchical cluster analysis of behavioral results of the MWMT using the protocol we previously reported [1,11,27]. Remarkably, we found that POCD mice showed abnormal behavioral performance in escape latency, platform crossing, and time spent in a target quadrant compared with non-POCD mice 1 week after anesthesia and surgery. At present, although several lines of evidence observed a significant increase in POCD symptoms approximately 3 days after anesthesia and surgery [28], we selected 1 week considering that POCD generally develops 1 week postoperatively.

α- and β-diversity are effective and practical indicators for the overall composition of gut microbiota [11,26]. The Shannon index was significantly decreased in POCD mice, whereas the Simpson index was markedly increased. This result indicates that the species and number of gut bacteria were significantly lesser in POCD mice than in non-POCD mice. β-diversity, including PLS-DA and PCoA, demonstrated that the dots of the non-POCD group were clearly separated from those of the POCD group. Recently, Yang et al. [29] reported that POCD could be alleviated using prebiotic galacto-oligosaccharide to target the gut–brain axis. These findings indicated that there was a significant dissimilarity in the composition of gut microbiota between the POCD and non-POCD phenotypes.

16S rRNA sequencing provides direct evidence on the role of specific bacteria in disease and treatment processes [30]. In this study, a total of 24 specific gut bacteria were significantly altered in the POCD versus the non-POCD phenotypes. Furthermore, 10 bacteria, including those from the *Chlamydiae* and *TM7* phyla, were increased in POCD mice than in non-POCD mice. *Mycoplasma* is a type of minimal prokaryotic cell that has no cell wall, is highly pleomorphic, and can be cultured in an artificial medium [31]. In this study, we found that the *Chlamydiae* phylum and *Chlamydiae* class were increased at five levels in the fecal samples of POCD mice. Interestingly, neurological symptoms occurred in approximately 25% of hospitalized pediatric patients with *Mycoplasma pneumoniae* infection [32]. More importantly, *Mycoplasma* infection preoperatively exacerbated the symptoms of POCD in 18-month-old rats [33], which is consistent with our results. It is therefore likely that *Mycoplasma* infection is highly associated with the onset and symptom severity of POCD. Treatment strategies such as macrolides antibiotics, which inhibit *Mycoplasma* infection, may favor recovery from POCD. Further detailed studies on the role of *Mycoplasma* in the pathogenesis and therapeutic mechanisms of POCD are clearly warranted.

*E. coli* is a  gram-negative,  facultative anaerobic, rod-shaped, coliform bacterium that is commonly present in the lower intestine of warm-blooded organisms [34]. In this study, we found that the abundance of *E. coli* was significantly increased in the feces of POCD mice. Although no study has reported on the role of *E. coli* in POCD, Barrientos et al. [35] found that *E. coli* has the pathological capacity to cause deficits in memory function in 24-month-old rats. Considering that β-lactam antibiotics are recommended to prevent and treat *E. coli* infection [36], it is implied that β-lactam antibiotics could be adopted to improve POCD. Large-scale clinical studies are required to validate this possibility in humans.

The increase in conditional bacteria can promote POCD, and the loss of several gut bacteria can also result in POCD. In this study, we demonstrated that the *Erysipelotrichia* class, *Erysipelotrichales* order, and *Erysipelotrichaceae* family, which belong to the *Firmicutes* phylum, were significantly decreased in the feces of POCD mice compared with that of non-POCD phenotype. Additionally, the *Anaeroplasmatales* order, *Anaeroplasmataceae* family, and *Anaeroplasma* genus, which belong to the *Tenericutes* phylum, were decreased in POCD mice. Collectively, these findings suggest that exogenous supplementation with *Firmicutes* and/or *Tenericutes* may be beneficial for the prevention and treatment of POCD.

As previously mentioned, although evidence based on peripheral blood biomarkers and CNS imaging have been reported to help confirm the diagnosis of CNS diseases [37,38], no objective and accurate indicators currently exist for the diagnosis of POCD, a condition that primarily depends on subjective cognition function scales. It is therefore likely that the objective quantification of the gut microbiota could provide a reference for the diagnosis and treatment of POCD. We first analyzed the correlation between gut bacteria levels and escape latency and found that a total of 13 gut bacteria were positively or negatively correlated with behavior. Next, we constructed ROC curves and found that the *Dehalobacteriaceae* family and *Dehalobacterium* genus may be sensitive indicators for the diagnosis of POCD, although their roles in POCD are still not well understood. Collectively, these findings suggest that the levels of gut bacteria could correlate with alterations in behavioral performance.

In conclusion, POCD is significantly associated with an abnormal composition of gut microbiota, and abnormalities in specific gut bacteria may be involved in the pathogenesis of POCD. The quantification of specific gut bacteria could provide objective indicators and new ideas for the diagnosis of POCD. Future clinical trials, however, are clearly needed to validate the role of gut microbiota in the pathogenesis and therapeutic mechanisms of POCD.

## MATERIALS AND METHODS

### Animals

A total of 20 eighteen-month-old male C57BL/6J mice (28–32 g) were obtained from the Laboratory Animal Center of Tongji Medical College, Huazhong University of Science and Technology (Wuhan, China). The animals were housed with a 12-h light/dark cycle and food and water ad libitum. The laboratory conditions were maintained at 22°C ± 2°C and a relative humidity of 60% ± 5%. All experimental protocols were performed in strict accordance with the National Institutes of Health guidelines and regulations. This study was approved by the Experimental Animal Committee of Tongji Hospital, Tongji Medical College, Huazhong University of Science and Technology (Wuhan, China).

### Anesthesia and surgery

As shown in Figure 1A, mice were acclimated to the environment for 7 days before the experiments were conducted. The intramedullary fixation for tibial fracture surgery was performed as previously described [39,40]. Briefly, mice were anesthetized using 3% isoflurane induction, followed by 2% isoflurane maintenance with 100% oxygen. Under aseptic surgical conditions, the left tibia was shaved and disinfected using povidone iodine. Next, a 0.3-mm pin was inserted into the tibial medullary cavity after the tibia was exposed, thus achieving intramedullary fixation, and osteotomy was performed. Finally, the incision was sutured with 5-0 Vicryl thread after necessary debridement, and compound lidocaine cream was applied to the wound locally twice daily for 3 days postsurgery for incision pain. The rectal temperature of the mice was maintained at 37°C ± 0.5°C during the surgery using a heating blanket. After surgery, the mice were placed back on the heated pads to recover and were then returned to their own cages with food and water *ad libitum*. The body weights of all mice were recorded.

### Open field test

As described in our previous study [1], each mouse was gently placed into the center of an open field chamber (40 × 40 × 40 cm) under dim light and allowed to move freely for 5 min. The movement parameters of all the mice were automatically monitored and analyzed by a video camera connected to the Any-Maze animal tracking system (Wuhan Yihong Technology Co., Ltd., Wuhan, China). The total distance covered was used to determine the locomotor activity of the mice undergoing anesthesia and surgery in this study.

### Morris water maze test

Spatial learning and memory function were assessed using the MWMT as reported in our previous study [11]. Mice were subjected to four trials each day for 5 consecutive days in a circular pool containing a 10-cm-diameter hidden platform, which was submerged 1 cm below the water surface in the target quadrant. Each mouse was permitted 60 s to find the submerged platform. If the mice failed to locate the platform, they were guided to the platform and remained there for 15 s before being returned to their cages. The time spent to reach the platform (escape latency) was recorded. On the sixth day, a 60-s probe trial was performed to assess reference memory, in which the platform was removed. The number of times the mice crossed the platform area (platform crossing) and time spent in the target quadrant were recorded using a digital video camera.

### 16S rRNA analysis of fecal samples

The fecal samples were collected immediately after all behavioral tests (Figure 1A). Samples were placed in 1.5-ml tubes, snap-frozen on dry ice, and stored at −80°C. The 16S rRNA analysis of fecal samples was performed by the Beijing Genomics Institute (Shenzhen, China). DNA extraction was performed using TIANamp stool DNA kits (Tiangen Biotechnology Company, Beijing, China). Genomic DNA was amplified in 50 μL triplicate reactions with bacterial 16S rRNA gene (V3–V4 region)-specific primers: 338F (5′-ACTCCTACGGGAGGCAGC-3′) and 806R (5′-GG ACTACHVGGGTWTCTAAT-3′). The reverse primer contained a sample barcode, and both primers were connected with an Illumina sequencing adapter. Polymerase chain reaction (PCR) products were purified, and the concentrations were adjusted for sequencing on an Illumina Miseq PE300 system. Original sequencing reads from the sample were sorted by unique barcodes, followed by removal of the barcode, linker, and PCR primer sequences. The resultant sequences were screened for quality, and ≥70 base pairs were selected for bioinformatics analysis. All sequences were classified using the National Center for Biotechnology Information BLAST and SILVA databases. Distance calculation, operational taxonomic units cluster, rarefaction analysis, and estimator calculation (α- and β-diversity) were performed using MOTHUR program [11].

### α-diversity analysis

α-diversity analysis, including Shannon and Simpson indices, were determined using MOTHUR ver.1.31.2. The Shannon and Simpson indices described the probability that the number of individuals obtained from the same two consecutive samples in a bacterium community [11].

### β-diversity analysis

β-diversity analysis was conducted as a heat map of Bray–Curtis diversity (calculated by QIIME ver.1.80) distance by using the heatmap function in the NMF package of R ver. 3.1.1.

### Partial least squares discrimination analysis

A PLS-DA analysis of the two principal components with the highest contribution was conducted using the mixOmics package of R ver. 3.1.1.

### Principal coordinates analysis

A PCoA analysis of Bray–Curtis was conducted using a random iterated algorithm in QIIME ver.1.80.

### Heat map analysis

Heat map analysis clustering of the relative abundance of six phylogenetic levels in all samples was conducted using Euclidean and complete functions in the gplots package of R ver. 3.1.1.

### Receiver operating characteristic curve

ROC curves illustrate the diagnostic ability of a binary classifier system with the true positive rate (sensitivity) as the ordinate and the false positive rate (1-specificity) as the abscissa. The ROC curves were used to detect the recognition of the gut bacterium to POCD. The closer the ROC curve is to the upper left corner, the higher is the accuracy. The value of the AUC (area under curve) represents the accuracy of the diagnosis.

### Statistical analysis

Values presented are expressed as mean ± standard error of the mean. The statistical analyses were performed using GraphPad Prism 7 (GraphPad Software, San Diego, CA, USA). Student’s *t*-test was used to measure the differences between the two groups. A correlation analysis was conducted using Pearson’s product-moment coefficient. The diagnostic cutoff values, sensitivity, specificity, and accuracy were determined by ROC curve analysis. *P* values <0.05 were considered statistically significant.
